
JOURNAL INFORMATION
==============================
NLM Title Abbreviation: ScientificWorldJournal
Journal ID: ScientificWorldJournal
NLM Journal ID: 101131163
Journal ID: TSWJ

The Scientific World Journal

ISSN: 2356-6140
EISSN: 1537-744X
Publisher: Wiley

ARTICLE INFORMATION
==============================
PMCID: PMC6084413
PMID: 30147535
DOI: 10.1100/tsw.2001.106
Article ID: 507865
Article version: 1
Subjects: Short Report

Cellular Senescence, Aging and Cancer

Campisi Judith
Lawrence Berkeley National Laboratory, Life Sciences Division, 1 Cyclotron Road, Mailstop 84-171, Berkeley, CA 94720, USA
Publication date: 2001
Electronic publication date: 2001 Apr 4
Volume: 1
First page: 65
Last page: 65
Copyright: Copyright © 2001 Judith Campisi.
Copyright year: 2001
License: This is an open access article distributed under the Creative Commons Attribution License, which permits unrestricted use, distribution, and reproduction in any medium, provided the original work is properly cited.
License URL: https://creativecommons.org/licenses/by/3.0/

Funding: National Institute on Aging

==============================
Miami *Nature Biotechnology* Short Reports
*TheScientificWorld* (2001) 1(S3), 67SR
ISSN 1532–2246; DOI 10.1100/TSW.2001.106

### CELLULAR SENESCENCE, AGING AND CANCER

Judith Campisi
Lawrence Berkeley National Laboratory, Life Sciences Division, 1 Cyclotron Road, Mailstop 84-171, Berkeley, CA 94720 USA
JCAMPISI@LBL.GOV

**INTRODUCTION**. Mammalian cells can respond to damage or stress by entering a state of irreversibly arrested growth and altered function termed cellular senescence. The senescence response can be elicited by one or more critically short telomere (replicative senescence), certain types of DNA damage or changes in chromatin structure, the expression of certain oncogenes, and supraphysiological mitogenic signals. Several lines of evidence suggest that cellular senescence evolved to suppress the development of cancer. Cellular senescence has also been proposed to contribute to aging, although strong evidence for this idea has been lacking.

We proposed that cellular senescence may be an example of antagonistic pleiotropy -- the idea that traits selected to benefit organismal fitness early in life can have unselected deleterious effects in aging organisms. The growth arrest associated with cellular senescence may be the selected trait that prevents the proliferation of cells that are at risk for neoplastic transformation. The altered functions associated with cellular senescence may be an unselected trait. The altered functions of senescent cells are cell-type specific, but often include secretion of molecules that can disrupt tissue microenvironments. Thus, the accumulation of dysfunctional senescent cells may contribute to the decline in tissue function and integrity that is a hallmark of aging. We also proposed that the accumulation of microenvironment-disrupting senescent cells may contribute to the exponential rise in cancer incidence that occurs with age.

**METHODS**. To test whether senescent stroma can stimulate preneoplastic epithelial cell growth, we cultured epithelial cells with presenescent or senescent human fibroblasts. We tested four epithelial cells: normal human keratinocytes, HaCAT human keratinocytes, S1 human mammary epithelial cells, and SCp2 mouse mammary epithelial cells. HaCAT, S1 and SCp2 are preneoplastic -- they are immortal and harbor p53 mutations, but do not form tumors in mice. We also cultured fibroblasts and epithelial cells in transwells, to exchange soluble factors without cell contact, and cultured epithelial cells on fibroblast extracellular matrices. In all cases, we monitored epithelial cell growth. Finally, we co-injected HaCAT, SCp2 or MDA-231 (a human breast cancer cell line) cells together with presenescent or senescent fibroblasts into nude mice, and monitored tumor formation and progression.

**RESULTS.** Senescent human fibroblasts stimulated the proliferation of preneoplastic HaCAT, S1, and SCp2 cells to a substantially greater extent than presenescent fibroblasts. By contrast, there was no difference between presenescent and senescent fibroblasts in their ability to stimulate the growth of normal keratinocytes. Approximately 50-60% (likely an underestimate)

of the senescence-associated stimulatory effects were due to insoluble (matrix) and soluble factors secreted by senescent cells. Senescent fibroblasts also markedly stimulated tumor formation (HaCAT, SCp2) and progression (MDA-231) in mice; many fewer and much smaller tumors were formed when the epithelial cells were co-injected with presenescent fibroblasts compared to senescent fibroblasts. The epithelial tumors that formed after co-injection with senescent fibroblasts were generally less differentiated and more invasive than those formed after co-injection with presenescent fibroblasts.

**DISCUSSION.** Our data support the idea that cellular senescence is an antagonistically pleiotropic trait -- protecting mammalian organisms from cancer early in life, but contributing to cancer incidence in older organisms. Senescent stromal cells secrete metalloproteinases, inflammatory cytokines and growth factors. The accumulation of senescent cells, particularly secretory senescent stromal cells, may synergize with mutation accumulation to promote epithelial tumor progression during aging.

**ACKNOWLEDGEMENT**. Supported by grants from the National Institute on Aging, DOD Breast Cancer Research Program, and UC Breast Cancer Research Program.
